# Nurses' perceptions of engaging in internet‐based nursing services: A qualitative study based on three hospitals in China

**DOI:** 10.1002/nop2.1934

**Published:** 2023-07-17

**Authors:** Baosheng Zhao, Wei Wang, Mo Yi, Hong LV, Xiaoman Zhang, Yujie Liu, Xinhong Song

**Affiliations:** ^1^ Shandong Provincial Hospital Affiliated to Shandong First Medical University Jinan Shandong China; ^2^ School of Nursing and Rehabilitation Shandong University Jinan Shandong China

**Keywords:** internet, nursing services, online nurses, perceptions, qualitative study

## Abstract

**Aim:**

In response to the ageing population and shortage of human resources for nursing care, China is piloting internet‐based nursing services (nurses who provide this care are called online nurses). Nurses are the providers of this model, so it is important to understand their perceptions. We aim to explore nurses' perceptions of engaging in internet‐based nursing services.

**Design:**

This study is descriptive qualitative research, so the data were analysed using a descriptive qualitative research method based on the theory of planned behaviour, using thematic analysis.

**Methods:**

With personal semi‐structured interviews conducted by two Master of Science in Nursing with 18 online nurses and nine clinical nurses, terminated after information saturation.

**Results:**

Nurses' emotional attitudes towards internet‐based nursing services were generally positive, but their behavioural intentions were negative. Social support, hospital organisational climate and family responsibilities had a statistically significant impact on nurses' behavioural decisions. Internet‐based nursing services place higher demands on nurses' knowledge and skills, and nurses are most concerned with ensuring patient and nurse safety.

**Patient or Public Contribution:**

No Patient or Public Contribution.

## 1 BACKGROUND

At present, China has the largest population of older individuals in the world, with approximately 165 million people aged 65 and over and 26 million people aged 80 and over at the end of 2019; as such, China must deal with the consequences of an ageing society for the foreseeable future (Fang et al., 2020; Wu et al., 2021). Key healthcare challenges stemming from an ageing population include the management of chronic noncommunicable diseases, cardiovascular disease, neurodegenerative diseases, physical frailty and mental health issues (Haidong Wang, 2016; Lancet, 2016). Patient demand for health care and services for older people continues to rise, and the burden on families and the public healthcare system continues to increase. Nurses play a pivotal role in the overall healthcare system; however, the shortage of nurses has been a worldwide health challenge (China, 2019a; Lancet, 2020). The questions of how to meet the growing demand for nursing services in the context of ageing and how to ensure successful ageing of older adults in the face of a nursing shortage are pressing challenges.

The development of the internet provides an excellent opportunity to reform and rethink the health and social security system of China, which is currently implementing internet‐based nursing services (nurses who provide this care are called online nurses; China, 2019b; China, 2019c). The hospital uses its own Registered Nurses and relies on the internet to provide care in the mode of ‘online application and offline service’. Patients place orders through the application (App; such as ‘Gold Nurse’, ‘Medical Care Home’, ‘Nurse Home’), Nurses take orders according to their qualifications and abilities and use their free time to address orders instantly. The management personnel choose the best matches for the orders via the information platform based on comprehensive factors, including nurses' qualifications, professionalism and distance. The nurses travel to patients' homes or designated locations by means of transportation, such as bicycles, cars, buses. They provide care services with respect to chronic disease management, rehabilitation care, special care, health education and hospice care for older or disabled patients, patients undergoing rehabilitation and terminal patients with limited mobility (China, 2019d). The care services currently provided include blood collection, intravenous infusion, intramuscular injection, subcutaneous injection, insurance services, general dressing change, catheterization, indwelling gastric tubes, peripherally inserted central venous catheters (PICC), medication exchange, enemas, suction, perineal care, stoma care, postnatal care and neonatal care (Huang et al., 2020; Ren et al., 2020).

Internet‐based nursing services are immediate, random and disposable, eliminating the time and space constraints of traditional care models, thereby maximising the human resources available for nursing and meeting the health needs of the population while increasing risks and challenges (Zhao et al., 2021; Zhao et al., 2022). Nurses are the main providers of internet‐based nursing services and only by fully understanding their views can targeted measures be taken to motivate them to participate and achieve the sustainability of the model (Hu, 2021). Relevant studies have already conducted cross‐sectional surveys of nurses' perceptions and attitudes (Huang et al., 2020; Li, Zhao, et al., 2020; Sheng et al., 2020). However, very little is currently known about the individual perceptions of nurses.

Theory helps to explain and predict behaviour. The theory of planned behaviour consists of three basic elements: attitudes, subjective norms and perceptual behavioural control (Ajzen, 1991; Godin & Kok, 1996). Attitudes refer to the positive or negative feelings that individuals experience with respect to adopting a particular behaviour and are conceptualised as an evaluation of that particular behaviour, such that attitudes are often viewed as a function of the individual's beliefs regarding the outcome of that behaviour. Subjective norms are social pressures that individuals feel with respect to their decision to adopt or not adopt a particular behaviour, and individuals or groups that influence behavioural decisions play an important role in determining whether individuals adopt a particular behaviour. Perceptual behavioural control is a reflection of an individual's past experiences and expected obstacles, and the more resources and opportunities an individual believes that he or she possesses and the fewer obstacles he or she expects, the greater that individual's perceptual behavioural control over behaviour.

The objective of this study was to explore nurses' perceptions of engaging in internet‐based nursing services based on three essential elements of the theory of planned behaviour. This study provides new insights into nurses' perceptions of engaging in internet‐based nursing services. These findings can inform future policy development and research directions.

## 2 METHODS

### 2.1 Design

The study used a descriptive, qualitative design and data were collected through semi‐structured individual interviews. We received Research Ethics Committee approval from Shandong Provincial Hospital Affiliated to Shandong First Medical University (22CGLJ36).

### 2.2 Study participants

This study was conducted in three general hospitals in China. Using a purposive sampling method, we recruited online nurses who had engaged in internet‐based nursing services for at least 6 months. To ensure that a wide range of attitudes and perspectives were represented, we selected online nurses according to the maximum variation strategy based on participant information such as age, gender, years of experience, job title and department, and we also included nurses who were eligible for online nurses but did not apply to participate. The study sample size was based on data saturation.

### 2.3 Data collection

The literature relevant to the purpose of the study was reviewed, and a preliminary outline of the interview was developed after thorough discussion among subject group members. Two nurses were selected separately for pre‐interviews, and the interview guidelines were then revised according to the results of these interviews to develop a formal interview outline (see Appendix A). Semi‐structured interviews were used to collect data, and times for the interviews were established by consulting the respondents in advance. The researcher established a good communication relationship with interviewees in advance via the hospital. The purpose and statistically significance of the study and the confidentiality of the interviews were explained before the interviews, and participants' consent was obtained by asking them to sign an informed consent form regarding the recording of the interview. The nurses were interviewed individually. This was a 2:1 interview. Two researchers interviewed one nurse, one asked questions while the other took brief notes. We conducted the interviews face‐to‐face. Two Master of Science in Nursing students conducted the interviews. All interviews were conducted by the researcher in a quiet and distraction‐free environment. During the interviews, the researcher listened carefully, maintained a neutral attitude, encouraged interviewees to express themselves fully, and observed and recorded their nonverbal communication. Each interview lasted 30–50 min, with the interviewer writing a reflective journal at the end of each interview to improve the quality of the interview. The interviews achieved data saturation after the 16th online nurse had been interviewed; two more online nurses were interviewed, and no new information emerged, resulting in a total of 18 online nurses interviewed. Semi‐structured interviews were conducted with corresponding clinical nurses, and data saturation was achieved by the seventh clinical nurse, with no new information emerging from interviews with two other clinical nurses, resulting in a total of nine clinical nurses interviewed.

### 2.4 Data analysis

Within 24 h of the interviews, two researchers independently transcribed the content of the audio recordings and returned these transcriptions to interviewees for confirmation of the interview content; subsequently, the data were analysed by two people simultaneously. The interview data and the audio recordings were read carefully and repeatedly, and the interviewees' words, expressions and movements were recalled in order to analyse and extract statistically significant content; recurring words were coded and identified, and themes were summarised and distilled; interviewees were consulted regarding points of disagreement for verification purposes. At the end of the data analysis, the subject group listened to the recordings once again to correct and review the preliminary analysis of the interview data and ensure the accuracy and precision of the analysis results. As this research is guided by a theoretical framework, data collection tends to be more structured than in the case of many other qualitative studies, and the analysis process tends to be more explicit. The analysis was conducted via thematic analysis (Braun & Clarke, 2006; Vaismoradi et al., 2013) and included the following steps: (1) familiarise yourself with the data: transcribe the data and read the data to jot down initial thoughts; (2) generate initial codes: systematically code interesting features of the data across the dataset, collating data associated with each code; (3) search for themes: collate the codes into potential themes and collect all data associated with each potential theme; (4) review the themes: verify that the themes are relevant to the coded extracts and the entire dataset, generating a theme map; (5) define and name the themes: perform ongoing analysis to refine the details of each theme and the overall narrative of the analysis, generating clear definitions and names for each theme; (6) produce a report: select vivid and engaging examples of extracts, conduct a final analysis of the selected extracts, relate the findings to the research questions and literature and generate an analysis report.

## 3 RESULTS

### 3.1 Participants

The basic characteristics of the participants are shown in Table 1. A total of 27 nurses were recruited, of whom 18 were online nurses and 22 were female. The average age of the participants was 34 years, and the average length of work was 10.6 years. Frequently provided services were intramuscular injection, subcutaneous injection, intravenous blood collection, urinary catheterization and indwelling gastric tubes.

**TABLE 1:** Basic characteristics of participants.

Number	Age	Gender	Educational level	Years of working experience	Job title	Department	Main services
N1	34	Female	Undergraduate	10	Charge Nurse	Intensive Care Unit	⑤⑥⑧⑨⑩
N2	30	Female	Undergraduate	7	Nurse Practitioner	Respiratory Medicine	①②③⑨
N3	41	Female	Undergraduate	16	Charge Nurse	Gastrointestinal Surgery	⑪
N4	31	Female	Specialty	9	Nurse Practitioner	Neonatology	⑬
N5	34	Female	Undergraduate	11	Charge Nurse	Urologic Surgery	②③⑤
N6	32	Male	Undergraduate	8	Nurse Practitioner	Emergency Department	①②③⑤⑥⑧
N7	42	Female	Masters	15	Deputy Chief Nurse	Medical Oncology	⑦
N8	30	Male	Undergraduate	7	Nurse Practitioner	Intensive Care Unit	①②③⑤⑥⑨⑩
N9	46	Female	Specialty	23	Charge Nurse	Cardiology	⑤⑥
N10	33	Female	Undergraduate	10	Charge Nurse	Endocrinology	⑭
N11	29	Female	Undergraduate	6	Nurse Practitioner	Obstetrics and Gynaecology	⑫
N12	30	Male	Specialty	9	Nurse Practitioner	Emergency Department	①②③⑤⑥
N13	31	Female	Masters	6	Charge Nurse	Breast Surgery	①②③⑥
N14	33	Female	Undergraduate	9	Nurse Practitioner	Medical Oncology	①②③⑦
N15	34	Female	Undergraduate	11	Charge Nurse	Intensive Care Unit	⑤⑧⑨⑩
N16	33	Female	Undergraduate	10	Nurse Practitioner	Gastroenterology	①②③⑥
N17	36	Female	Specialty	15	Charge Nurse	Orthopaedics	④
N18	35	Female	Undergraduate	11	Charge Nurse	Emergency Department	①②③⑤⑥
N19	34	Female	Masters	8	Charge Nurse	Emergency Department	
N20	33	Female	Undergraduate	9	Nurse Practitioner	Orthopaedics	
N21	35	Female	Specialty	12	Nurse Practitioner	Endocrinology	
N22	32	Female	Undergraduate	8	Nurse Practitioner	Medical Oncology	
N23	34	Male	Undergraduate	11	Charge Nurse	Intensive Care Unit	
N24	35	Female	Specialty	13	Charge Nurse	Obstetrics and Gynaecology	
N25	41	Female	Undergraduate	16	Charge Nurse	Neonatology	
N26	32	Male	Specialty	9	Nurse Practitioner	Emergency Department	
N27	31	Female	Undergraduate	7	Nurse Practitioner	Gastrointestinal Surgery	

*Note*: (1) N1–N18 are online nurses, and N19–N27 are clinical nurses. (2) ① Venous blood collection; ② intramuscular injection; ③ subcutaneous injection; ④ surgical stitch removal; ⑤ urinary catheterization; ⑥ indwelling gastric tube; ⑦ PICC medication change; ⑧ enema; ⑨ sputum aspiration; ⑩ pressure injury care; ⑪ stoma care; ⑫ postnatal care; ⑬ neonatal care; ⑭ diabetic foot medication change.

### 3.2 Themes

#### 3.2.1 Attitudes

Nurses' emotional attitudes towards internet‐based nursing services were generally positive, but nurses' behavioural intentions were negative (Table 2). Nurses said that internet‐based nursing services met the diverse health needs in the context of ageing and fulfilled the attachment of older patients to their homes. It reduces the time of patients' hospital visits and the risk of in‐transit, especially during a regular epidemic, and the model greatly reduces the risk of cross‐infection. In addition, the project can improve the professional identity of nurses and broaden their career paths. However, the working model of internet‐based nursing services conflicts with the recreational life of nurses, and some nurses fear being stigmatised by the pornographic industry. Coupled with insufficient publicity and popularisation, some nurses do not understand or even misunderstand the specific content, such as nursing service authority, nursing service form, remuneration. In addition, internet‐based nursing services are in a pilot stage, immature and with many risks, for example, road safety, the prevention of occupational violence, and the treatment of critical condition changes caused by home nursing procedures and nurses' willingness to act are shown to be neutral and wait‐and‐see.

**TABLE 2:** Attitudes.

Theoretical framework	Themes	Subthemes	Quotes
Attitudes	Emotional attitude is positive	Meeting a diverse range of health needs	N9: ‘As people's living standards improve and their health needs become more diversified *[…]* internet‐based care services riding on the internet express are an effective way to cope with ageing and are a sunrise industry’.
		Satisfying the attachment of older patients to their homes	N15: ‘Influenced by traditional Chinese culture *[…]* older or terminally ill patients want to be treated at home during the last days of their lives, which will increase their sense of well‐being’.
		Reducing hospital visit times and transfer risks	N1: ‘Patients are bedridden and have limited mobility, hospital visits are time consuming in terms of human and material resources, they need to be physically tortured during transfers, they bear the risk of wind chill, sudden conditions and the risk of infection during epidemics *[…] i*nternet‐based care services solve these problems’.
		Improving the professional identity of nurses	N12: ‘Home visits are more likely to be recognised and accepted by patients and families. In hospital they take it for granted that nurses provide a service, but with this home visit, they have a special respect for you and their sense of value is multiplied *[…] o*n my arrival, I see the expectation and gratitude of the patients’.
		Broadening career paths for nurses	N6: ‘Nurses can take orders at any time, working with autonomy and flexibility, taking orders according to their own time and wishes *[…]* providing a new pathway for nurses' career development, which can be developed as a specialist direction’.
	Behavioural intention is negative	Conflict with recreational life	N22: ‘Nursing work is busy, with little time off, it is already physically and mentally exhausting, I would rather go and watch a movie or play badminton than use my spare time to provide nursing care *[…]* after all, work is for life’.
		Stigmatised by the sex industry	N1:: ‘Nurses, mostly young women, provide nursing services to patients through home visits, some operations involve patients' private parts *[…]* and some comments on the internet link internet‐based nursing services to the sex industry’.
		Insufficient publicity and misconceptions	N20: ‘What are the requirements for nurses, how to apply, what nursing services are provided and other specifics are not known *[…]* I thought the model was mainly for critically ill patients, like an ambulance’.
		Start‐up phase, immature project	N19: ‘Currently in the trial stage, policies, laws and other aspects are still immature, such as safety and security, incentives, service standards are not yet clear *[…]* when the model is mature I will consider applying to become an online nurse’.

#### 3.2.2 Subjective norms

The subjective norms of nurses are influenced by three areas: social support, hospital organisational climate and family responsibilities (Table 3). Currently, there is a lack of legislation related to internet‐based nursing services, insufficient research on policy content and economic conflict between nurses and patients. In addition, internet‐based nursing services are not sufficiently motivating for hospitals, hospitals place less importance on them, nurses lack a voice in leadership, chief nursing officers and colleagues have indifferent attitudes, nurses want internet‐based nursing services to be integrated with the performance appraisal system and promotion system, and they expect assistance from multidisciplinary teams to support and give full play to the role of primary nurses. In addition, younger nurses are more willing to participate in internet‐based nursing services to subsidise their families, but this conflicts with the qualification requirements in the policy. For middle‐aged and older nurses, internet‐based nursing services conflict with the family roles that nurses play as wives, mothers and daughters.

**TABLE 3:** Subjective norms.

Theoretical framework	Themes	Subthemes	Quotes
Subjective norms	Social support	Lack of relevant legislation	N25: ‘The legality of multipoint practice for nurses, whether nurses have prescriptive authority in emergency situations *[…]* the determination of illegal practice of medicine, and the handling of medical disputes are unclear’.
		Insufficient research on policy content	N13: ‘Managers and researchers in nursing need to focus on service standards, service lines, indicators for evaluating quality of care *[…]* accreditation systems, training programmes, emergency plans, security and regulation of online platforms’.
		Nurse and patient in financial conflict	N3: ‘Currently, internet‐based care services are not covered by health insurance, patients have a greater financial burden *[…]* financial income does not provide a greater incentive for nurses, which may require government subsidies’.
	Hospital organisational	Insufficient incentives for hospitals	N7: ‘Although the hospital is actively pursuing it out of social responsibility *[…]* after all, the project is not profitable for the hospital and bears all kinds of risks, which is not enough incentive for the hospital’.
		Lack of voice of nurses in leadership	N9: ‘Hospital leadership, mostly doctors, are not as knowledgeable about the model as nurses and do not pay enough attention to it *[…] n*eed for nurses to have a voice in leadership’.
		Apathetic attitude of head nurse and colleagues	N14: ‘The head nurse in our department is concerned about distraction and the impact on her job *[…] s*ome colleagues are not very motivated, and individual colleagues see orders being taken and give negative comments’.
		Looking forward to having the support of a multidisciplinary team to assist	N5: ‘The catheter had been successfully inserted and the patient had not been able to urinate and the patient was in distress *[…] i*t took a phone call to a urologist I know for advice to get the problem sorted out’.
		Integrated with the performance appraisal and promotion system	N19: ‘Apart from their own work, nurses focus more on writing articles and declaring topics because it is closely related to promotion *[…]* and only by linking internet‐based care services to performance and promotion will more nurses be attracted to join’.
		Making the most of the role of the primary nurse	N16: ‘Internet‐based care services should be more often undertaken by nurses from secondary or community hospitals *[…]* but due to their lack of capacity, they are currently mostly nurses from tertiary hospitals’.
	Family responsibilities	Conflicting wishes and qualifications of young nurses	N7: ‘For middle aged and older nurses who meet the qualification requirements, who have worked for many years and are financially stable, this salary is not attractive enough, younger nurses need this salary more *[…]* but younger nurses do not meet the policy requirement of 5 years' experience’.
		Conflict with family roles	N16: ‘Every time I take an order during my time off, my husband will drive me, which means warmth and guilt *[…]* I need to play the multiple roles of wife, mother and daughter, they are the most important in my heart and I need to spend more time off to be with them and take care of them’.

#### 3.2.3 Perceptual behavioural control

Nurses possessed solid basic knowledge and conventional nursing skills, but internet‐based nursing services placed higher demands on nurses' knowledge and skills, and ensuring the safety of nurses and patients was a core concern of nurses (Table 4). The unfamiliar and rudimentary home environment greatly increased the difficulty of nursing operations, and nurses emphasised the importance of communication skills and emergency response abilities, arguing that online nurses need to be uniformly trained and certified. In addition, there is a risk to breaching confidentiality of patients' personal data and health information, and there are many patient safety risks due to the distance from standardised wards, the fact that the source of medicines and medical consumables has not been standardised and the lack of resuscitation equipment and medicines. At present, the personal safety and transport safety of nurses cannot be guaranteed and there is an urgent need to improve patient identification and accident insurance for nurses.

**TABLE 4:** Perceptual behavioural control.

Theoretical framework	Themes	Subthemes	Quotes
Perceptual behavioural control	Knowledge and skills	Solid basic knowledge and routine nursing skills	N12: ‘Having worked in acute care for so many years *[…]* I am comfortable with the basic theory and skills to meet the professional requirements of internet‐based care services’.
		The home environment makes it statistically significantly more difficult to operate	N8: ‘Mostly unfamiliar patients, bedridden for long periods of time, unable to take care of themselves, the mood of the patients and their families was poor and the level of cooperation from the patients was not good, I was nervous throughout the home visit *[…]* the home environment put higher demands on the nursing operation’.
		Emphasis on communication skills and emergency response	N2: Communication is important, sometimes you operate in general, but you are good at communicating and patients are willing to give you another chance even if you make a mistake *[…]* and the ability to deal with emergencies because there is more uncertainty in the family’.
		Uniform training and qualification	N24: ‘It is essential to be uniformly trained and certified by a professional body before taking orders *[…]* and certification serves as a mechanism to protect patients and ensure that competent nurses enter practice to improve the quality of health care’.
	Patient Safety	Away from standardised wards, increasing medical risks	N13: ‘There was no suitable place for medical items in the nursing operation, on several occasions chairs were used as treatment trolleys, some patients were in shaded rooms and the lights were not lit, the hardware was poor *[…]* there were many safety hazards’.
		Sources of medicines and medical consumables not yet harmonised	N6: ‘The source of medicines and medical consumables is unclear, with some patients providing them themselves and others requiring medical staff to carry them *[…]* making their quality and safety difficult to guarantee’.
		Emergency resuscitation is not guaranteed	N7: ‘If a patient has a medical emergency, it is difficult to ensure patient safety due to the lack of resuscitation drugs and resuscitation instruments and the lack of support from the medical team *[…]*, which is the core problem’.
		Breaching confidentiality of patients' personal and health information	N1: ‘Online platforms have detailed personal and health information of patients, such as disease history, past history and diagnosis *[…]*, which cannot be protected by patients' rights if they are compromised’.
	Nurse safety	Personal safety hazards	N4: ‘Nurses are mostly female and work alone, facing unfamiliar environment and unfamiliar patients, if a nurse–patient dispute occurs, without the assistance of colleagues or security guards, we have to face it alone, one click to call the police *[…]* and wait for the police to arrive may have been harmed’.
		Traffic safety hazards	N2: ‘Once I went to a patient's home, the patient lived in a remote area, it was raining and the roads were bad, so I rode my own bike *[…]* a traffic safety hazard of concern’.
		Improve patient identification and nurse accident insurance	N4: ‘The accreditation of patients' identities and home visits for initial home visits should be improved, the safety and potential risks of the service should be accurately assessed *[…]* and the state should purchase liability, medical accident and personal accident insurance for nurses’.

## 4 DISCUSSION

Our study found that nurses' affective attitudes towards internet‐based nursing services were generally positive, with nurses believing that internet‐based nursing services, which have been introduced in the context of an ageing population and which make use of the internet under governmental guidance, and the shift from a hospital to a home care model met patients' diversified health needs, enhanced nurses' professional mission and identity and broadened nurses' career development paths. However, nurses' willingness to act was negative, and nurses believed that the model is still in a pilot phase, lacking laws and policies, receiving insufficient attention from hospitals and departments, providing insufficient incentives, leading to family role conflicts, lacking training programmes and featuring hidden risks to patient safety, among other issues that must be resolved.

Currently, internet‐based nursing services lack strong legal protections (Liu et al., 2019). The rights and responsibilities of hospitals, nurses, patients and third‐party platforms should be defined clearly in terms of medical disputes and how to manage emergencies. Provisions should be made clearly and enforced strictly with respect to disputes, the preservation of rights and attributions of responsibility. China has not fully liberalised multipoint practice for nurses, and nurses do not have prescription rights (Huang et al., 2019; Li, Liu, et al., 2020; Yin & Li, 2017). China can learn from countries such as the United States and Australia (Carryer et al., 2007; Dunn, 1997), which have gradually and carefully liberalized multipoint practice for nurses and provided certain prescription rights to nurses with master's degrees or above and specialist nurses. The pricing of care services should take into account the interests of all three parties and can draw on data from countries such as Japan (Iwagami & Tamiya, 2019; Tamiya et al., 2011) to include those parties in the medical insurance reimbursement catalogue. This approach would allow for the establishment of a sound, long‐term care insurance system to regulate a reasonable price range and ensure that online nurses are motivated while reducing patients' medical expenses. In terms of policy, the government has proposed specific and rigid requirements for hospitals, which should assume social responsibility and respond positively to the national health reform system. In addition, administrators and researchers focused on nursing should improve the certification system for online nurses, care service items, care service processes and standards, quality evaluation index systems, emergency plans and other policy elements as soon as possible.

Our study found that online nurses had negative experiences, such as experiences of lack of behavioural motivation, lack of supportive unit leadership and lack of team support. Good organisational fit and supportive and caring colleagues and managers enhance the commitment of nurses to their work (Cao et al., 2021). Therefore, nursing managers should create a good organisational atmosphere, schedule shifts appropriately, formulate a reasonable employment mechanism, appropriately regulate labour intensity so that nurses have the time and energy to coordinate their work role with their family and life roles, and guide network nurses to expand their professional values on the basis of improving their own balance between work and life. In addition, managers should integrate performance appraisal systems and promotion systems with internet‐based nursing services to motivate middle‐aged and older nurses to engage in internet‐based nursing services in a more positive way.

The randomness, immediacy and disposability of internet‐based nursing services can maximise the use of nursing human resources, but the specificity of the service site and the unpredictability of the population and the emergencies they face also increase safety risks for nurses and patients (Xie, 2019; Zhao et al., 2021). Such services should be demand‐driven and refined in terms of theoretical knowledge, operational skills, safety awareness, ethics and regulations, communication and etiquette, operational environment, use of items and equipment, medical consumables and medical waste disposal, risk warning awareness, rapid response capability and first aid techniques; in addition, such services should seek to develop practical, scientific and quantitative training programmes to improve the professionalism and qualifications of nurses (Shen et al., 2021; Xv et al., 2020; Zhao et al., 2022).

Currently, nurses are predominantly female and work alone. If they are unable to accurately grasp patients' personality traits and assess the characteristics of their social support system, in addition to the fact that patients may have high treatment expectations or perceptions of the connection between medical care and nursing, the occupational or even personal safety of nurses can be difficult to guarantee in the event of medical disputes or medical incidents (Han et al., 2020; Wang et al., 2019). For patients making their first appointment, the hospital and the platform must first confirm and assess their illness, operation needs, identity and service location and confirm the service distance, service time and service location involved before sending the online nurse to provide the relevant care service. In addition, it is necessary to ensure that the location of online nurses is tracked accurately and that police officers are able to respond in a timely manner after an duress device is triggered; it is also important to purchase personal accident and medical accident insurance for online nurses (Hu et al., 2019).

## 5 LIMITATIONS

This study faces several limitations. First, all respondents were nurses from three tertiary general hospitals in Shandong Province, so the results may not be generalisable to other provinces and lower‐level hospitals. Second, we did not interview staff from other units regarding their perceptions of internet‐based nursing services, such as doctors and staff from third‐party platforms. Finally, it is critical to explore patients' perceptions of internet‐based nursing services directly and to investigate whether patient experiences of internet‐based nursing services and their potential limitations and benefits are consistent with those expressed by nurses.

## 6 CONCLUSIONS

This study explored nurses' perceptions of engaging in internet‐based nursing services based on three basic elements of the theory of planned behaviour. Our study found that nurses' affective attitudes towards internet‐based nursing services were generally positive, with nurses believing that internet‐based nursing services, which were introduced in the context of an ageing population and which make use of the internet under governmental guidance, and the shift from a hospital care model to a home care model met patients' diversified health needs, enhanced nurses' professional mission and sense of identity and broadened nurses' career development paths. However, nurses' willingness to act was negative, with nurses believing that the model is still in a pilot phase and that problems such as a lack of laws and policies, a lack of incentives, family role conflicts and hidden risks to patient safety still need to be addressed. These findings can influence future policy development and research directions. Future research should focus on ways of addressing the challenges faced by nurses who participate in internet‐based care services.

## AUTHOR CONTRIBUTIONS

Baosheng Zhao and Wei wang was responsible for designing the study, conducting the interviews, interpreting the data and drafting the text. Mo yi was responsible for conducting the interviews and interpreting the data. Hong Lv was responsible for interpreting the data. XiaoMan Zhang was responsible for interpreting the data. Yujie Liu was responsible for interpreting the data. Xinhong Song was responsible for designing the study, critical review of the intellectual content of the article, support and guidance. All authors reviewed the manuscript.

## CONFLICT OF INTEREST STATEMENT

I declare that the authors have no competing interests as defined by Nursing Open or other interests that might be perceived to influence the results and/or discussion reported in this paper.

## ETHICS STATEMENT

We obtained approval from the Ethics Committee of Shandong Provincial Hospital Affiliated to Shandong First Medical University.(2021259).

## Data Availability

The data that support the findings of this study are available on request from the corresponding author. The data are not publicly available due to privacy or ethical restrictions.

## APPENDIX 1

### 1.1 INTERVIEW OUTLINE

#### A.1 Interview Outline 1: (Online Nurse)

(1) How do you feel about internet‐based care services? What are your reasons for engaging in it? In what ways does it make a difference?

(2) What mode of transport do you usually use to provide internet‐based care services? What are the main services provided? How do you feel during the care service?

(3) What do you think is the difference between care services provided in hospitals and internet‐based care services?

(4) What do you think is the level of support and acceptance of internet‐based care services from hospitals, patients and society?

(5) What difficulties have you encountered by participating in internet‐based care services? How were they resolved?

(6) What qualities and competencies do you think online nurses must have?

(7) What are your expectations regarding the emerging product of internet‐based care services? What are your recommendations?

#### A.2 Interview Outline 2: (clinical nurse)

(1) Have you heard of internet‐based care services? How do you feel about internet‐based care services?

(2) What are your reasons for not getting involved? Will you be involved in the future?

(3) What difficulties do you think could be encountered in the implementation of internet‐based care services?

(4) What qualities and competencies do you think online nurses must have?

(5) What are your expectations regarding the emerging product of internet‐based care services? What are your recommendations?

#### A.3 General probes

(1) Can you explain this in detail?

(2) Can you think of an example?

(3) How do you feel about it?

(4) What do you suggest?

(5) Do you have anything else to add?
